# Appraisal of the faecal haemoglobin, age and sex test (FAST) score in assessment of patients with lower bowel symptoms: an observational study

**DOI:** 10.1186/s12876-019-1135-5

**Published:** 2019-12-11

**Authors:** Jayne Digby, Judith A. Strachan, Craig Mowat, Robert J. C. Steele, Callum G. Fraser

**Affiliations:** 1 Centre for Research into Cancer Prevention and Screening, University of Dundee, Dundee, Scotland UK; 2 Blood Sciences and Scottish Bowel Screening Laboratory, Ninewells Hospital and Medical School, Dundee, Scotland UK; 3 Department of Gastroenterology, Ninewells Hospital and Medical School, Dundee, Scotland UK; 4 Centre for Research into Cancer Prevention and Screening, University of Dundee, Dundee, Scotland UK; 5 Centre for Research into Cancer Prevention and Screening, University of Dundee, Ninewells Hospital and Medical School, Dundee, Scotland DD1 9SY UK

**Keywords:** Adenoma, Bowel disease, Colorectal cancer, Faecal biomarkers, Faecal haemoglobin, Faecal immunochemical test, FAST score, Inflammatory bowel disease

## Abstract

**Background:**

Many patients present in primary care with lower bowel symptoms, but significant bowel disease (SBD), comprising colorectal cancer (CRC), advanced adenoma (AA), or inflammatory bowel disease (IBD), is uncommon. Quantitative faecal immunochemical tests for haemoglobin (FIT), which examine faecal haemoglobin concentrations (f-Hb), assist in deciding who would benefit from colonoscopy. Incorporation of additional variables in an individual risk-score might improve this approach. We investigated if the published **f**-Hb, **a**ge and **s**ex **t**est score (FAST score) added value.

**Methods:**

Data from the first year of routine use of FIT in primary care in one NHS Board in Scotland were examined: f-Hb was estimated using one HM-JACKarc FIT system (Kyowa Medex Co., Ltd., Tokyo, Japan) with a cut-off for positivity ≥10 μg Hb/g faeces. 5660 specimens were received for analysis in the first year. 4072 patients were referred to secondary care: 2881 (70.6%) of these had returned a FIT specimen. Of those referred, 1447 had colonoscopy data as well as the f-Hb result (group A): 2521 patients, also with f-Hb, were not immediately referred (group B). The FAST score was assessed in both groups.

**Results:**

1196 (41.7%) of patients who returned a specimen for FIT analysis had f-Hb ≥10 μg Hb/g faeces. In group A, 252 of 296 (85.1%) with SBD had f-Hb > 10 μg Hb/g faeces, as did 528 of 1151 (45.8%) without SBD. Using a FAST score > 2.12, which gives high clinical sensitivity for CRC, only 1143 would have been referred for colonoscopy (21.0% reduction in demand): 286 of 296 (96.6%) with SBD had a positive FAST score, as did 857 of 1151 (74.5%) without SBD. However, one CRC, five AA and four IBD would have been missed. In group B, although 95.2% had f-Hb < 10 μg Hb/g faeces, 1371 (53.7%) had FAST score ≥ 2.12: clinical rationale led to only 122 of group B completing subsequent bowel investigations: a FAST score > 2.12 was found in 13 of 15 (86.7%) with SBD.

**Conclusions:**

The performance characteristics of the FAST score did not seem to enhance the utility of f-Hb alone. Locally-derived formulae might confer desired benefits.

## Background

Faecal immunochemical tests (FIT) for haemoglobin (Hb) are widely used in opportunistic and programmatic screening for colorectal cancer (CRC) in asymptomatic populations [1]. However, most cases of CRC in the United Kingdom (UK), and probably elsewhere, are diagnosed after referral from primary to secondary care [2]. A difficulty in practice is that very many patients present with lower bowel symptoms in primary care, but significant bowel disease (SBD: colorectal cancer [CRC], advanced adenoma [AA], sometimes precursors of CRC, or inflammatory bowel disease [IBD]) is uncommon. Symptoms have been well documented to have poor diagnostic accuracy for SBD [3, 4]. However, the faecal haemoglobin concentration (f-Hb), as determined by quantitative FIT, has been proven to be of considerable value in the assessment of symptomatic patients [5–7]. The National Institute for Health and Care Excellence (NICE) in England has developed diagnostic guidance (DG30) [8] which states that: quantitative FIT are recommended for adoption in primary care to guide referral for suspected colorectal cancer in people without rectal bleeding who have unexplained symptoms but do not meet the criteria for a suspected cancer pathway referral outlined in NICE’s guideline on suspected cancer (NG12) [9]. DG30 also states that results should be reported using a threshold (cut-off) of 10 μg Hb/g faeces [8]. However, a number of very real challenges still confound the introduction of FIT in assessment of patients presenting in primary care with lower bowel symptoms [10], which include rectal bleeding, a change in bowel habits, weight loss, anaemia, abdominal pain and blood in faeces [8].

It has been proposed that prediction or risk-scoring models, which combine symptoms and/or known risk factors for SBD with f-Hb, might improve the use of f-Hb alone [11]. A CRC prediction model, COLONPREDICT, based on both clinical and laboratory findings, was developed by Cubiella et al., who compared its diagnostic accuracy with the 2015 version of the NICE NG12 referral criteria and externally validated the strategy: it was concluded that COLONPREDICT was a highly accurate prediction model for CRC detection [12]. The final prediction model included 11 variables and, in consequence, we thought that this complexity might make this approach unlikely to be used in routine primary care practice. Since we have demonstrated that f-Hb is affected by age and sex, with f-Hb rising with age and being higher in men than women [13], an international multi-centre collaboration led to development and validation of the FAST score, which combines **f**-Hb and **a**ge and **s**ex as a single test result which might indicate individual risk of CRC and SBD [14]. The developers considered that the FAST score was easy to calculate and was highly accurate for CRC detection in symptomatic patients. Interestingly, the validation cohort included heterogeneous data from three studies in Scotland as well as two studies in different regions of Spain, using a number of different FIT analytical systems, and it appeared that the FAST score was equally clinically sensitive for CRC, regardless of country, prevalence of disease, age, sex, healthcare level (primary or secondary) and analytical system used to estimate f-Hb. However, it was admitted that the diagnostic accuracy and applicability of the FAST score in a primary care setting had still to be investigated objectively.

Recently, a comprehensive diagnostic accuracy study was performed in Spain with data from the 1572 patients in the COLONPREDICT cohort [15]. The conclusion was that referral criteria based on f-Hb, used either on its own or as a component of two prediction models, COLONPREDICT and the FAST score, are more accurate than symptom-based referral criteria for CRC detection in patients presenting with lower bowel symptoms. The symptom-based criteria examined included those currently used in the UK 2017 update of the NICE NG12 strategy [9], which was disseminated following publication of NICE DG30 [8]. However, it is not known to date whether use of the FAST score, rather than f-Hb along with clinical impressions and perhaps the use of other routine tests including the full blood count, would add value in different geographical settings and using different criteria for referral for colonoscopy and different FIT analytical systems. This study aimed to perform an initial evaluation of the utility of the FAST score in patients presenting in primary care for investigation of lower bowel symptoms, in one region of Scotland, over the first year of routine use of FIT.

## Methods

Data from the first year of routine use of f-Hb in NHS Tayside, Scotland, were included in our examination of the use of the FAST score: the full details of the evaluation of the impact of introducing a FIT for into primary care on the outcome of patients with new bowel symptoms have been published [16]. In brief, since December 2015, general practitioners (GP) were encouraged to request a FIT when evaluating patients presenting with new lower bowel symptoms. FIT kits, comprising one specimen collection device (Kyowa Medex Co., Ltd., Tokyo, Japan) along with pictorial instructions and a return envelope, were provided to patients by general practitioners (GP). When a FIT sample was returned to the laboratory, f-Hb was estimated using one HM-JACKarc FIT system (Kyowa Medex). Samples with results above the upper analytical limit were not diluted and re-analysed but reported as ≥400 μg Hb/g faeces. Results with f-Hb < 400 μg Hb/g faeces but ≥10 μg Hb/g faeces were defined as positive and reported numerically. The cut-off f-Hb was as recommended in NICE DG30 [8]: results < 10 μg Hb/g faeces were reported as f-Hb not detected. The reports also directed GP to the NHS Tayside gastroenterology website, which advised that f-Hb < 10 μg Hb/g faeces, in the absence of iron deficiency anaemia (IDA), rectal bleeding, or a mass, suggests that SBD is extremely unlikely. All requests for further investigation in secondary care were made through a unique electronic portal. Age and sex were determined from the Community Health Index (CHI), a unique 10-digit patient identifier used throughout primary and secondary care in NHS Scotland.

**Fig. 1:**
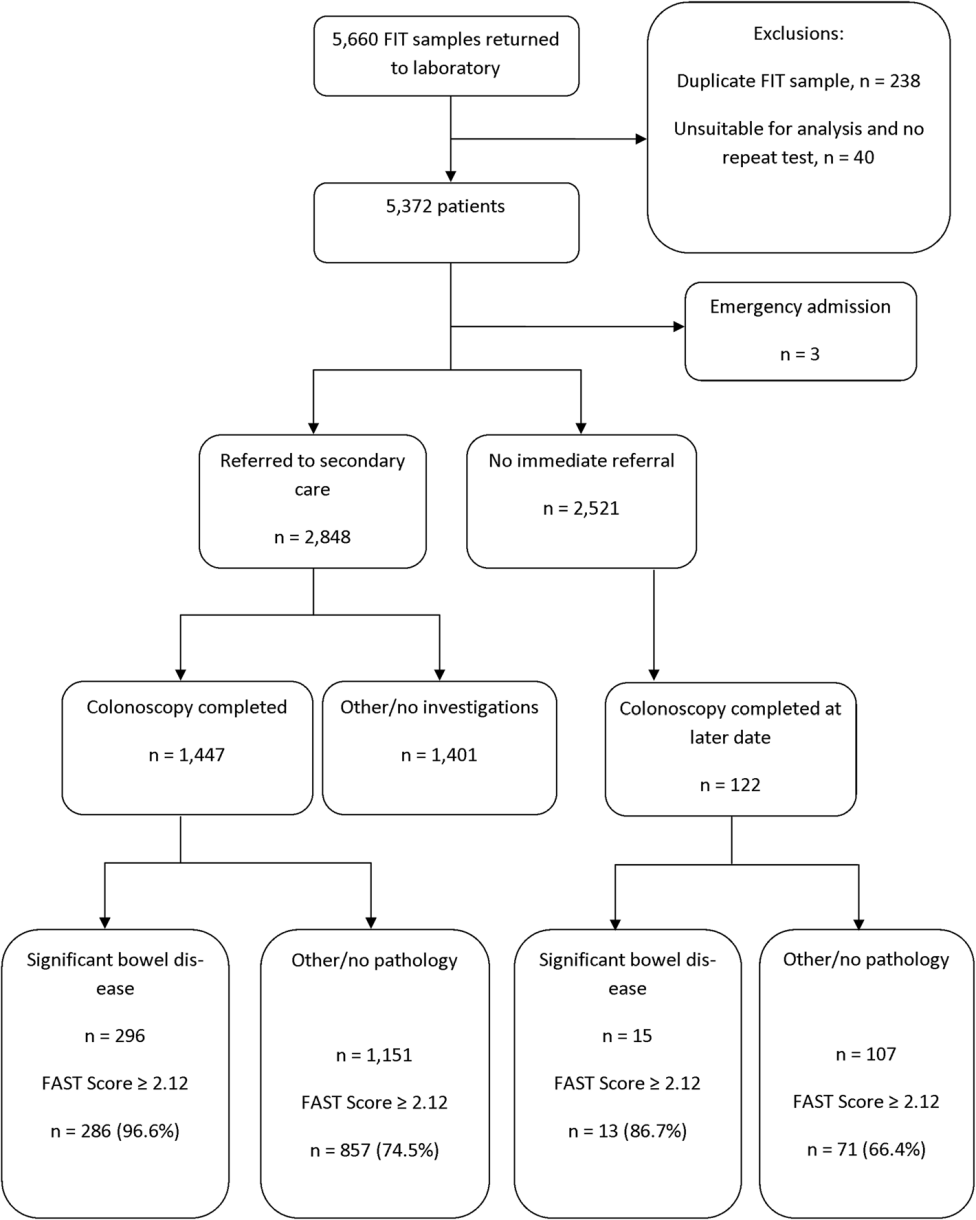
Study flow diagram

Five thousand six hundred sixty specimens were received in the first year for f-Hb examination (Fig. 1). Further investigation in secondary care was requested via the portal for 4072 patients and 2881 of these returned a specimen for f-Hb examination: at the end of the first year, 1447 patients had completed a FIT and had undergone colonoscopy in secondary care (group A). The utility of the FAST score was examined in this group A.

2521 patients who had submitted FIT samples were not associated with an immediate referral. The potential utility of the FAST score in this group B was also assessed and outcomes for patients followed for approximately one further year to assess whether subsequent referral had occurred and whether SBD had been missed. In addition, linkage with the Scottish Cancer Registry was performed to ensure that all cases of CRC had been identified in both groups A and B.

The FAST score was calculated as detailed earlier using logistic regression, following a univariate analysis using generalized additive models with smoothing splines for continuous variables. Regression coefficients which were used to construct a CRC prediction score, where the dependent variable was the presence or absence of CRC [14]. For f-Hb 0 μg Hb/g faeces, f-Hb score = 0, if f-Hb from 1 to 19 μg Hb/g faeces, score = 0.684, if f-Hb from 20 to 199 μg Hb/g faeces, score = 2.824 and, if f-Hb > 200 μg Hb/g faeces, score = 4.184.Then, the FAST score = f-Hb score + 0.031 x age (years) + 0.479 if male. Interpretation is: if the FAST score is ≥2.12, there is a high risk of CRC; a score of < 2.12 implies that there is little risk of CRC and referral for colonoscopy would be of limited value. The threshold for the β-coefficient of the FAST score with 99% clinical sensitivity was 2.12 and was at least that in 88.0% of the patients included in the derivation cohort.

MedCalc statistical software (MedCalc Software, Mariakerke, Belgium) was used for all calculations. All data were gathered within routine patient care, therefore ethical approval was not required.

## Results

Group A comprised 1447 patients with new bowel symptoms who completed a FIT and were referred to secondary care and for whom colonoscopy data, and histology if required, were available: 780 (53.9%) of these had f-Hb > 10 μg Hb/g faces. There were 296 with SBD of whom 252 had a positive f-Hb result (f-Hb > 10 μg Hb/g faeces) and 1151 without SBD of whom 623 had a negative f-Hb result (f-Hb < 10 μg Hb/g faeces). Using a FAST score with > 2.12 as the cut-off so as to give 99% clinical sensitivity for CRC according to its developers [14], only 1143 of the 1447 would have been referred for colonoscopy, a 21.0% reduction in colonoscopy demand. Of the 296 with SBD, 286 had a positive FAST score. Of the 1151 without SBD, only 294 had a negative FAST score. The ten cases detected by f-Hb and other information, but not by the FAST score, were one CRC, five AA and four IBD. Further investigation of referral data for these cases revealed that the patient with CRC was referred due to iron deficiency anaemia (IDA). Three of the five AA reported persistent diarrhoea, one had rectal bleeding and the remaining AA was a lesion which had been palpated upon digital rectal examination by the GP. Of the four cases of IBD associated with a negative FAST score, two reported rectal bleeding and two had a change in bowel habit.

The number of colonoscopies required, number (percentage) with CRC and SBD, and positive predictive value, negative predictive value, sensitivity and specificity, with 95% confidence intervals (CI), for both CRC and SBD in the 1143 patients in group A with FAST score > 2.12 are shown in Table 1.

**Table 1:** Outcomes for 1143 patients with FAST score > 2.12 in 1447 referred patients with both faecal haemoglobin concentration and colonoscopy results

Outcome	Colorectal cancer (CRC) in the 1143 with FAST score > 2.12	Significant bowel disease (SBD) in the 1143 with FAST score > 2.12
No. of colonoscopies	1143	1143
No (%) with disease	94 (8.2)	286 (25.0)
Positive predictive value (%) with (95% CI)	8.2 (8.0–8.5)	25.0 (24.3–25.8)
Negative predictive value (%) with (95% CI)	98.9 (97.7–100.0)	96.7 (94.1–98.2)
Sensitivity (%) with (95% CI)	99.0 (94.3–100.0)	96.6 (93.9–98.4)
Specificity (%) with (95% CI)	22.4 (20.2–24.7)	25.5 (23.1–28.2)

Of the 2521 in group B who were not immediately referred from primary care for colonoscopy, only 4.8% had f-Hb *>* 10 μg Hb/g faeces. In marked contrast, 1349 (53.5%) had FAST score ≥ 2.12. The majority of these patients with negative f-Hb results were not investigated further. Indeed, clinical rationale led to only 122 (4.7%) of these patients ultimately completing bowel investigations. There were 15 cases of SBD subsequently diagnosed: four cases of CRC, five cases of AA and six new cases of IBD. A FAST score ≥ 2.12 was found in 13 (86.7%) of these, including all four CRC, which were associated with an initial f-Hb < 10 μg Hb/g faeces, as were three of the five AA and three of the six new cases of IBD.

## Discussion

The published FAST score was derived from a cohort consisting of 1572 consecutive symptomatic patients referred to colonoscopy who were included in the derivation cohort for the COLONPREDICT study. The validation cohort included data from three studies done in Scotland and two in Spain. It was shown that a FAST score < 2.12 implied that CRC can be ruled out. This would facilitate the major aim of our use of FIT in assessment of patients presenting with lower bowel symptoms, which is as a “rule-out” test, thereby directing the somewhat limited colonoscopy resource available to those who would benefit most and, more importantly, reassuring those with a low FAST score that the likelihood of SBD was low. However, safety-netting approaches, including watching and waiting, should be in place for those who continue to complain of symptoms [10]. In addition, it was acknowledged that the patients included in all of the studies included in the derivation and validation of the score had been selected a priori by health care professionals for further evaluation. Further, it was stated that it was considered that the diagnostic accuracy of the FAST score should be externally evaluated in patients presenting in primary care with lower bowel symptoms before its use was extensively adopted. The recent work of Herrero et al. [15] demonstrated that 88.0% of the 1572 in the COLONPREDICT cohort studied had a FAST score ≥ 2.12: thus, few (only 12.0%) would be deemed to have a low risk of CRC and not require colonoscopy. The clinical sensitivity for the FAST score > 2.12 was 100.0% (95% CI: 97.8–100.0) and 93.7% (95% CI: 95.9–98.9) for CRC and SBD respectively, and the specificity was 13.9% (95% CI: 12.1–15.9) and 16.1% (95% CI: 14.0–18.4). However, a major caveat is that the data used, from the COLONPREDICT study, were those used as the derivation cohort for development of the FAST score.

In contrast, our analysis of the application of the FAST score in patients referred from primary care with lower bowel symptoms, on whom both f-Hb and colonoscopy data were available, demonstrated that the colonoscopy demand would be reduced using a FAST score ≥ 2.12 cut-off. The clinical performance characteristics for CRC and SBD are shown in Table 1. The sensitivity and specificity in group A were lower and higher, respectively, from those found in the recent study of Herrero et al. on the use of the FAST score [15]. This might be because their group has significant differences to ours, particularly the higher prevalence of CRC and SBD. Eight cases of SBD would have been missed if the FAST score > 2.12 was used as compared to f-Hb plus clinical data, but only one case of CRC, which would have been referred anyway and not missed clinically, since the patient had IDA. However, a limitation of our study is that group A had been referred to secondary care without use of the FAST score: thus, in this study, the use of the FAST score has actually been examined as a potential follow-up investigation prior to acceptance into colonoscopy, assessing whether the score, applied after referral on the grounds of clinical findings and f-Hb, would lead to a reduction in colonoscopies: it does, but a small number of patients with CRC and SBD would be missed, a less than ideal finding. Further examination of the cases of SBD which would be missed if the FAST score had been applied showed the importance of clinical rationale, because all 15 had what some would term “red flag” symptoms. However, similar symptoms were also reported by the majority of the remaining 294 referred patients who also had a negative FAST score, but did not have SBD; 96 had IDA, 78 had rectal bleeding, 46 had persistent diarrhoea and 47 had a change in bowel habit. A prospective study, using the FAST score as the initial triage tool in routine practice therefore seems to be warranted from both our data and those of Herrero et al. [15]

In those in group B, who were not referred, possibly in the main because 95.0% had f-Hb < 10 μg Hb/g faeces, over 50% had a FAST score > 2.12 and thus, if this became the criterion for referral, many more colonoscopies would be required, without much evidence of the value of further investigations in this group. One concern, worthy of further examination, is that the current score means that all males over 53 years would be referred as would all females over 68 years and this is the major reason why the score has such high positivity in patients in group B that were not referred by GP for further investigation. However, clinical rationale led to only 122 (4.7%) being further investigation over the subsequent year. Interestingly, despite f-Hb < 10 μg Hb/g faeces, the FAST score again detected all four CRC found in this group. It also would have detected all five AA and four out of six IBD, but the specificity would be very low. A clear limitation of this study is that colonoscopy was not performed on all of group B, but this reflects real routine practice. Subsequent linkage with the Scottish Cancer Registry confirmed that all patients with CRC in this group were identified.

## Conclusions

As a result of this study, we cannot support use of the published FAST score formula [15] to assist in deciding who to refer for colonoscopy, since SBD was missed in those who had been referred using f-Hb and other considerations: further, the positivity was more than 50% in those not referred. In the original development of the FAST score, it was admitted that a limitation was the reduced diagnostic accuracy for detection of not only CRC, but additional SBD, possibly because the covariate pattern which predicts CRC may be different to that which predicts other bowel diseases. On the other hand, although this reduced accuracy could be considered as a limitation, there are several cogent arguments to support use of the FAST score in this broader clinical perspective [14]. However, we believe that the score could be improved. One major concern is that the published score has f-Hb groups based on the analytical performance characteristics of the OC-Sensor (Eiken Chemical Co., Ltd., Tokyo, Japan), which has an analytical working range of 10–200 μg Hb/g faeces, and the 20 μg Hb/g faeces used in the formula was selected during development as the most commonly applied f-Hb cut-off in CRC screening. We used a different FIT analytical system. Albeit that our original development and validation of the FAST score suggested that there were no inter-analyser differences [10], we think that a FAST score with f-Hb groups of < 10, 10–400 and > 400 μg Hb/g faeces should be created for the FIT system most widely used in the UK for assessment of symptomatic patients (HM-JACKarc, Kyowa Medex Co., Ltd., Tokyo, Japan): groups such as this are required because f-Hb does not have a normal distribution, even after logarithmic transformation, and these groups are used in our and others’ current routine application of FIT in routine practice. This concept, that different FIT analytical systems may require different FAST score formulae, is supported by the results from a recent study [17]. A comparison showed that the new COLONFIT score, which includes the maximum f-Hb of three samples and the number of samples with f-Hb > 4 μg Hb/g faeces in addition to other variables, classified patients 3–4% better than the FAST score in both the derivation and validation cohorts. It was suggested that further studies on a direct comparison of both scores are needed to assess if the 3–4% gain in classification could be offset by lower adherence through the requirement for three faecal samples rather than one. Importantly, however, the FIT analytical system (iFOB, Linear Chemicals SL, Barcelona, Spain), which was said to be able to detect f-Hb of 4 μg Hb/g faeces, was different to that used to derive the FAST score and that used in this study. The performance of the FAST score with this FIT analytical system perhaps could have been improved as suggested above, but with different and appropriate f-Hb groups.

Moreover, it may be that there is benefit in using lower f-Hb cut-offs than the 10 μg Hb/g faeces recommended in DG30 [8], for example, using the limit of detection (2 μg Hb/g faeces for the FIT analytical system used in this study) or the limit of quantitation (7 μg Hb/g faeces) [18]. Although we have recently suggested that f-Hb is the most important factor to be considered when deciding which patients presenting in primary care with lower bowel symptoms would benefit most from referral for colonoscopy [19], we plan to create a number of scores and examine the use of these including lower f-Hb cut-offs derived from the detectability characteristics of the FIT system used, which do differ between FIT systems [18]. Ideally, this should be done prospectively in our now routine use of FIT in assessment of most patients presenting in primary care [16], other than those with rectal bleeding, IDA, or a mass, although the application of the FAST score does warrant further study in such patients, who are currently deemed to be at high risk. In addition, further risk scores that include f-Hb and other variables, should be developed and investigated prospectively.

## Data Availability

The datasets used and analysed during the current study are available from the corresponding author on reasonable request.

## Abbreviations

AA: advanced adenoma
CHI: Community Health Index
CRC: colorectal cancer
DG: diagnostics guidance
f-Hb: faecal haemoglobin concentration
FIT: faecal immunochemical test for haemoglobin
GP: general practitioner
IDA: iron deficiency anaemia
NG: NICE guideline
NICE: National Institute for Health and Care Excellence
SBD: significant bowel disease
UK: United Kingdom
